# Human Milk as a Biomonitor of Toxic Metal Exposure: Sources, Transfer Mechanisms, and Implications for Infant Health—A Review

**DOI:** 10.3390/nu18101527

**Published:** 2026-05-12

**Authors:** Danuta Katryńska, Agnieszka Bzikowska-Jura, Zofia Goc, Łukasz Kogut

**Affiliations:** 1 Department of Zoology, Faculty of Exact and Natural Sciences, University of the National Education Commission, 30-084 Krakow, Poland; danutakatrynska@gmail.com; 2 Department of Clinical Dietetics, Faculty of Health Sciences, Medical University of Warsaw, 00-575 Warsaw, Poland; abzikowska@wum.edu.pl; 3 Department of Animal Physiology, Faculty of Exact and Natural Sciences, University of the National Education Commission, 30-084 Krakow, Poland; zofia.goc@uken.krakow.pl; 4 Department of Bioenergetics, Food Analysis and Microbiology, Institute of Food Technology and Nutrition, Faculty of Technology and Life Science, University of Rzeszów, Aleja Rejtana 16C, 35-959 Rzeszów, Poland

**Keywords:** human milk, breastfeeding, heavy metals, environmental exposure, maternal–infant transfer, toxic elements, biomonitoring

## Abstract

Human milk (HM) is recognized as the optimal source of nutrition for infants, providing essential nutrients, bioactive compounds, and immunological protection crucial for proper growth and development. However, due to increasing environmental pollution, HM may also serve as a vector for exposure to toxic substances, including heavy metals. These contaminants originate from both current environmental exposure and long-term accumulation in maternal tissues, which may be mobilized during pregnancy and lactation. **Objectives:** The aim of this review was to comprehensively analyze the occurrence, sources, and determinants of heavy and toxic metals in human milk, with particular emphasis on maternal–infant transfer pathways and geographical variability of exposure. **Methods:** A structured narrative review with systematic literature search elements was conducted using PubMed, Scopus, and Web of Science databases. The search covered studies published between 2010 and 2025 and was limited to articles written in English. The search strategy included terms related to human milk and heavy metal exposure (Pb, Cd, Hg, As, Cr, Al). Predefined inclusion and exclusion criteria were applied, and a qualitative synthesis of environmental, dietary, physiological, and lifestyle-related determinants, as well as geographical variability, was performed. **Results:** The available evidence indicates that heavy metals are commonly detected in human milk worldwide, with concentrations strongly influenced by environmental pollution, maternal diet, and lifestyle factors. Under typical exposure conditions, reported concentration ranges are approximately 2–5 µg/L for lead (Pb), 1.4–1.7 µg/L for mercury (Hg), and below 1 µg/L for cadmium (Cd). However, substantially higher levels have been reported in highly contaminated regions, with extreme values exceeding 1000 µg/L for Pb and 100 µg/L for Hg in isolated cases. Key exposure pathways include contaminated food, drinking water, air pollution, and endogenous mobilization of metals stored in maternal tissues (particularly bone and adipose tissue). Significant geographical variability was observed, with higher concentrations reported in industrialized and mining regions. Infants represent a highly vulnerable population due to immature detoxification systems, increased gastrointestinal absorption, and ongoing neurodevelopment, which may amplify toxic effects even at low exposure levels. **Conclusions:** Although human milk remains the gold standard for infant nutrition, the presence of heavy metals highlights the need for continuous environmental monitoring and preventive strategies aimed at reducing maternal exposure. The benefits of breastfeeding clearly outweigh the potential risks; however, minimizing environmental contamination remains a critical public health priority. Future research should focus on standardizing analytical methods, improving biomonitoring strategies, and better characterizing long-term health outcomes associated with early-life exposure to toxic metals.

## 1. Introduction

Environmental and dietary factors may contribute to elevated exposure to harmful substances during critical developmental windows, potentially affecting maternal lactation and increasing infant susceptibility to toxicants [1]. The World Health Organization (WHO) recommends extending the period of breastfeeding to two years, and in the first six months it should be the sole and obligatory nutrition of the baby [2]. This recommendation is supported by medical associations such as the American Academy of Pediatrics (AAP) and the European Society for Paediatric Gastroenterology, Hepatology and Nutrition (ESPGHAN) [3,4].

In addition to nutrients, vitamins and antioxidants, human milk (HM) also contains fewer undesirable molecules such as xenobiotics. Xenobiotics are synthetic chemicals that can be found in every organism but are not produced by the organism itself [5]. This review integrates evidence on multiple toxic elements to provide a unified analysis of environmental exposure pathways, maternal body burden, transfer into human milk, and infant exposure during lactation. The human population is chronically exposed to very low concentrations of xenobiotics due to continuous exposure to consumer chemicals. The latter include personal care products and cosmetics (e.g., parabens), disinfectants (e.g., triclosan), plant protection products (e.g., herbicides, insecticides, biocides), pharmaceutical drugs, plastics (e.g., phthalates, bisphenols) and many others used in industrial processes and as components of other products (e.g., toxic and heavy metals) [6]. Hence, people are exposed to xenobiotics mainly through the food chain, so they are referred to as “food pollutants” [7,8,9]. Diet modification and variable consumption during the perinatal period can lead to epigenetic effects—molecular phenomena that affect gene expression without altering nuclear DNA (mutation) [10]. Exposure to environmental pollutants and dietary xenobiotics from conception to the end of fetal life is crucial as such developmental periods have been described as critical developmental windows. This concept describes how disruptions during the prenatal period can cause chronic or endocrine diseases later in life [11,12,13]. Human milk provides all the macro- and microelements in the diet as well as hundreds of non-nutrients, bioactive molecules necessary for the survival and well-being of an infant and for its proper growth and development [14,15]. Milk can therefore be a carrier of water- and fat-soluble contaminants [16].

In this review, we have focused on heavy and toxic metals (arsenic, cadmium, lead, chromium, mercury, and aluminum), as they represent an important and potentially harmful group of environmental contaminants. This review provides an integrated and critical synthesis of current evidence of heavy metal contamination in human milk, with particular emphasis on the relationship between environmental exposure, maternal body burden, and transfer to the infant. This work builds on existing literature by integrating data on multiple toxic elements and providing a combined analysis of environmental, dietary, and physiological determinants, as well as geographical variability in exposure [17].

**Figure 1:**
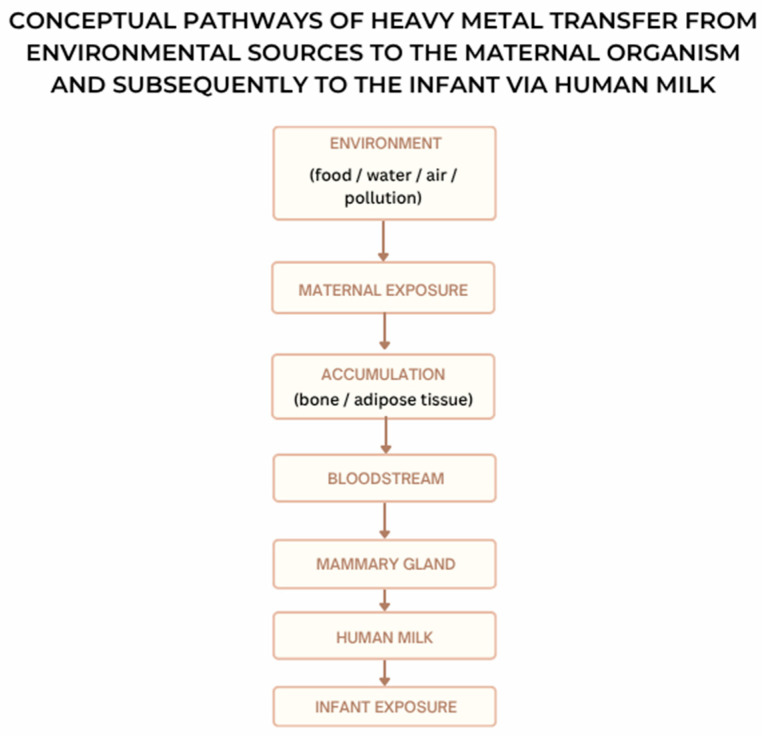
Conceptual pathways of heavy metal transfer from environmental sources to the maternal organism and subsequently to the infant via human milk.

To provide a structured analytical framework, the present review follows a pathway-based approach, linking environmental sources of heavy metals with maternal exposure, transfer into human milk, and subsequent infant exposure (Figure 1). This framework enables a clearer interpretation of how environmental contamination translates into measurable levels of toxic elements in human milk.

This review provides a structured and integrative synthesis of available evidence rather than a quantitative assessment of effect size.

Although previous reviews have addressed the presence of heavy metals in human milk, the present study provides an integrated perspective combining geographical variability, maternal–infant transfer mechanisms, and determinants of exposure within a unified analytical framework.

The scheme illustrates the main exposure routes, including contaminated food, water, and air, leading to maternal accumulation in tissues (bone and adipose tissue), followed by mobilization into the bloodstream and transfer to the mammary gland, ultimately resulting in infant exposure through breastfeeding.

## 2. Materials and Methods

This review was conducted as a structured narrative review with systematic literature search elements. Although a structured search strategy, predefined inclusion criteria, and a defined publication timeframe (2010–2025) were applied, the review was not designed as a formal systematic review and did not include protocol registration, formal risk-of-bias assessment, or meta-analysis. The aim of the methodology was to ensure transparent and reproducible literature identification while maintaining the interpretative and integrative character of a narrative review.

The search strategy combined keywords and controlled vocabulary (MeSH terms where applicable) related to human milk and heavy metal exposure, including the following query structure: (“human milk” OR “breast milk”) AND (“heavy metals” OR “toxic elements”) AND (“lead” OR “cadmium” OR “mercury” OR “arsenic” OR “aluminum” OR “chromium”) AND (“exposure” OR “transfer” OR “biomonitoring” OR “contamination”). The search strategy was adapted for each database using a combination of free-text keywords and controlled vocabulary. Boolean operators (AND, OR) were used to combine search terms, and minor modifications were applied depending on database-specific indexing systems.

Studies were included if they were original research articles or review papers assessing the presence or concentration of heavy metals in human milk, conducted on lactating women, and addressing environmental, dietary, physiological, or lifestyle-related determinants of exposure. Studies were excluded if they were conducted exclusively on animals, lacked quantitative or relevant descriptive data, were conference abstracts, editorials, or non-peer-reviewed publications, or if full-text access was unavailable.

The initial database search yielded approximately 520 records. After removing duplicates, titles and abstracts were screened for relevance, resulting in 148 articles selected for full-text evaluation. Following the application of inclusion and exclusion criteria, a total of 112 studies were included in the final qualitative synthesis.

Data were extracted regarding study characteristics, including geographical location, study population, analyzed metals, reported concentration ranges, and determinants of exposure. A qualitative synthesis approach was applied to identify common patterns, sources of variability, and major pathways of maternal–infant exposure to heavy metals via human milk.

To strengthen the critical integration of evidence, the included studies were additionally evaluated in terms of methodological robustness, including study design, sample size, analytical technique, and control of confounding factors. Greater interpretative weight was given to studies employing validated high-sensitivity analytical methods (e.g., ICP-MS), larger sample sizes, and clearly defined sampling protocols. In contrast, findings from studies with limited methodological detail or small sample sizes were interpreted with caution. Furthermore, differences between environmental, epidemiological, and experimental studies were considered when drawing conclusions, recognizing that these approaches provide complementary but not equivalent levels of evidence.

The methodology followed principles of transparent literature searching and study selection; however, the present work was conducted as a structured narrative review rather than a formal systematic review. Therefore, PRISMA reporting standards were not fully applied.

For clarity and improved interpretability of the evidence, the extracted studies were additionally organized into structured summary tables according to geographical region, sample size, lactation stage, analytical method, and reported concentration ranges, enabling semi-quantitative comparison across studies.

It should be acknowledged that, due to the narrative nature of the review, the search strategy may not have captured all relevant studies, and selection bias cannot be fully excluded.

## 3. Heavy Metals Relevant to Human Milk: Sources, Maternal Exposure, and Infant Transfer

Although environmental sources of heavy metals are briefly outlined to provide context, the primary focus of the following sections is on their relevance to maternal exposure, their transfer into human milk, and their role as a pathway of infant exposure. In this context, human milk is treated as a dynamic biological matrix that reflects both current exposure and the mobilization of previously accumulated contaminants, enabling a more targeted interpretation of early-life exposure. Detailed environmental and plant-related mechanisms are beyond the scope of this review and are discussed only to the extent necessary to contextualize maternal exposure pathways.

Heavy metals originate from both natural and anthropogenic sources, leading to their accumulation in environmental compartments such as soil, water, and air. Human exposure occurs primarily through contaminated food, drinking water, and inhalation, contributing to maternal body burden and subsequent transfer into human milk [17,18,19,20,21,22,23,24].

From the perspective of human exposure, diet represents one of the most important routes of heavy metal intake. Plants readily absorb both essential and non-essential elements from the soil, including toxic metals such as arsenic (As), cadmium (Cd), chromium (Cr), mercury (Hg), and lead (Pb), which may subsequently accumulate in edible plant tissues [25,26,27]. As a result, the consumption of plant-based foods, particularly cereals, vegetables, and rice cultivated in contaminated soils, constitutes a major pathway of exposure for the human population [25,26,27,28,29,30]. In addition to dietary intake, heavy metals may also enter the body through contaminated water and inhalation of polluted air [21,22,23,24].

Although the mechanisms of metal uptake and accumulation in plants have been extensively described [31,32,33,34], their relevance in this context lies primarily in their contribution to human exposure. Long-term intake of contaminated food and water leads to the accumulation of metals in the maternal organism, where they may be stored in various tissues or circulate in the bloodstream. During pregnancy and lactation, physiological changes may promote the mobilization of these elements and facilitate their transfer to the developing fetus and, subsequently, to the infant via human milk.

Therefore, environmental processes such as soil contamination, plant uptake, and food chain transfer should be considered as upstream determinants of maternal body burden and, consequently, of heavy metal concentrations in human milk. Understanding these pathways is essential for assessing infant exposure during breastfeeding and for identifying key points of intervention to reduce health risks [35].

### 3.1. Arsenic (As)

Arsenic (As) is a metalloid commonly present in the environment and characterized by high toxicity potential. In countries such as Bangladesh, China, Hungary, and India, As occurs at high concentrations in groundwater and surface soils [36]. In addition to its natural occurrence, As is also released into the environment as a result of smelting and mining processes, agricultural practices, and the production and use of preservatives and food additives [37]. Arsenic is considered a major public health concern due to its toxic and carcinogenic properties [38,39].

Human arsenic exposure occurs predominantly through contaminated drinking water and dietary intake, particularly rice and cereal-based products [39,40,41,42,43,44,45,46,47,48]. Long-term dietary exposure may contribute to systemic accumulation of arsenic in the maternal organism and subsequently influence its transfer into human milk during lactation [42,43,44,45].

In addition to plant-based foods, arsenic exposure may also occur through seafood consumption, although it is predominantly present in less toxic organic forms [49,50,51]. Overall arsenic exposure is influenced by dietary habits, environmental contamination, and food preparation practices, which may affect its chemical speciation and bioavailability [51,52]. Consequently, long-term dietary exposure represents a key determinant of arsenic accumulation in the human body [48,53].

In the maternal organism, chronic exposure to arsenic may lead to its systemic distribution and accumulation, influenced by metabolic processes such as methylation. Arsenic is capable of crossing biological barriers and has been detected in human milk, indicating that exposure may continue during lactation. The concentration of arsenic in breast milk reflects both current environmental exposure and internal redistribution processes [52,53].

Infant exposure to arsenic via human milk may be biologically relevant due to the high susceptibility of the developing organism. Early-life exposure has been associated with potential effects on neurodevelopment, immune function, and epigenetic regulation. Therefore, environmental and dietary sources of arsenic should be considered as key determinants of maternal body burden and subsequent infant exposure during breastfeeding [48,49,50,51,52,53].

### 3.2. Cadmium (Cd)

Cadmium contamination is primarily associated with industrial emissions, phosphate fertilizers, and contaminated irrigation water [54,55]. These processes result in the accumulation of Cd in soils and its subsequent transfer into crops, thereby introducing this element into the food chain.

Diet represents the primary route of cadmium exposure, particularly through cereals, vegetables, and rice cultivated in contaminated soils [56]. Dietary cadmium exposure may be particularly relevant in populations relying on locally produced food from contaminated regions [56,57,58]. Long-term dietary exposure contributes to cadmium accumulation within the maternal organism and may subsequently influence its presence in human milk during lactation [57,59,60,61,62,63,64,65].

Long-term dietary exposure to cadmium contributes to its gradual accumulation in the human body, where it is transported in the form of Cd–protein complexes, primarily bound to metallothioneins, and stored mainly in the liver and kidneys with a biological half-life of up to 10 years [66,67,68]. The cumulative nature of cadmium exposure may be particularly relevant in women of reproductive age, as maternal body burden reflects long-term environmental and dietary exposure.

During pregnancy and lactation, physiological changes may influence the mobilization and redistribution of cadmium within the maternal organism. Although the transfer of Cd into human milk is relatively limited compared to other heavy metals, its presence has been documented and reflects chronic maternal exposure.

Cadmium exposure via breast milk, even at low levels, may be relevant due to the immature detoxification and excretory systems in early life. Infants may therefore be more susceptible to cadmium accumulation and its potential toxic effects, including impacts on renal function and development. Consequently, environmental and dietary sources of cadmium should be considered key determinants of maternal body burden and subsequent infant exposure during breastfeeding [65,68].

### 3.3. Lead (Pb)

Lead (Pb) is a widespread environmental contaminant with no known physiological function in biological systems. Its presence in the environment is largely associated with anthropogenic activities, including metal smelting, industrial processes, agriculture, and commercial emissions [69,70,71]. As a result, lead accumulates in soil, water, and air, from which it can enter the food chain and contribute to human exposure. Chronic lead exposure has been associated with adverse effects involving multiple organ systems, particularly the central nervous and cardiovascular systems.

From the perspective of human exposure, ingestion of contaminated food and water and inhalation of particulate matter represent the main pathways of lead intake [72,73,74]. Although plant uptake of lead is generally limited compared to other metals, contamination of crops may still occur, particularly in polluted environments, thereby contributing to dietary exposure [75,76,77,78]. Dietary intake and inhalation represent important pathways of chronic lead exposure [79,80].

One of the major toxicokinetic features of lead is its long-term accumulation in the human body, particularly in bone tissue, where it may persist for decades. In the bloodstream, lead binds to proteins and low-molecular-weight compounds, facilitating its distribution to various organs. Importantly, during pregnancy and lactation, increased calcium demand leads to bone resorption, resulting in the release of previously stored lead into the circulation. This endogenous source of exposure is particularly relevant in women of reproductive age [81].

Lead is capable of being transferred into human milk, and its concentration reflects both current environmental exposure and long-term accumulation in maternal tissues. Consequently, breastfeeding may contribute to infant lead exposure, particularly under conditions of elevated maternal body burden, even in the absence of ongoing environmental contamination [72,80,81].

From the perspective of infant health, exposure to lead during early life is of particular concern due to the high sensitivity of the developing nervous system. Even low levels of exposure have been associated with neurodevelopmental impairment, cognitive deficits, and behavioral disturbances. Therefore, environmental and dietary sources of lead should be considered key determinants of maternal body burden and subsequent infant exposure via human milk [72,80,81].

### 3.4. Chromium (Cr)

Chromium (Cr) is an environmental contaminant occurring both naturally and as a result of anthropogenic activities, including electroplating, tanning, pigment production, and industrial emissions. Chromium exists in several chemical forms differing in toxicity and bioavailability, with hexavalent chromium considered the most toxic [74,82,83].

Human exposure to chromium occurs mainly through contaminated food, drinking water, and, in some cases, inhalation of polluted air. The toxicity and bioavailability of chromium depend largely on its chemical form, with Cr (VI) being more readily absorbed and capable of inducing oxidative stress, DNA damage, and adverse effects on multiple organ systems [74,82,83,84,85].

Dietary intake represents an important pathway of chromium exposure, particularly in areas affected by environmental contamination. Human chromium exposure occurs mainly through contaminated food, drinking water, and industrial pollution, contributing to systemic distribution and potential accumulation within the maternal organism [86,87,88,89,90,91,92,93].

Maternal chromium exposure appears to be influenced by both environmental and dietary factors. Its presence in the body may result in systemic distribution, and transfer into human milk has been reported, although available data are limited and show variability depending on exposure conditions. Nevertheless, this indicates that chromium may contribute to postnatal exposure during breastfeeding [74,82,83].

Exposure to chromium via human milk may be relevant due to the vulnerability of the developing organism and limited detoxification capacity. Potential biological effects may include oxidative stress and alterations in metabolic processes, particularly in the case of exposure to more toxic forms such as Cr (VI). Therefore, environmental and dietary sources of chromium should be considered as factors influencing maternal body burden and subsequent infant exposure through lactation [74,82,83,92,93].

### 3.5. Mercury (Hg)

Over the past two centuries, emissions of mercury (Hg) have increased significantly due to anthropogenic activities, particularly from coal combustion and chlor-alkali industry processes [94]. As a result, mercury is widely distributed in the environment and can enter soil, water, and the food chain, representing a major source of human exposure. Mercury is widely recognized as a high-priority toxic element due to its documented effects on human health [95,96,97].

Dietary intake, particularly through fish and seafood consumption, is regarded as the dominant route of mercury exposure [98,99]. As a result, higher trophic level organisms, including fish consumed by humans, may contain elevated concentrations of mercury [100].

In the human organism, mercury is absorbed through the gastrointestinal tract and distributed to various tissues, with a particular affinity for the central nervous system. The toxicity of mercury depends on its chemical form, with organic mercury compounds, especially methylmercury, being the most harmful due to their high bioavailability and ability to cross biological barriers [100,101].

In the maternal organism, mercury exposure primarily reflects dietary habits, especially fish consumption. Mercury is capable of crossing the placental barrier and has also been detected in human milk, indicating that exposure may occur both prenatally and during lactation. Consequently, breast milk may represent a route of postnatal exposure to mercury in infants [97,98,99,100,101].

Early-life mercury exposure is considered particularly relevant due to the susceptibility of the developing nervous system to neurotoxic effects. Early-life exposure, especially to methylmercury, may impair brain development, leading to cognitive deficits, motor dysfunction, and long-term neurological consequences. Therefore, environmental and dietary sources of mercury should be considered key determinants of maternal body burden and subsequent infant exposure via breastfeeding [100,101].

### 3.6. Aluminum (Al)

Aluminum is a widespread environmental contaminant originating from both natural and anthropogenic sources [102,103,104,105,106,107]. In addition to natural sources, human exposure to aluminum is largely driven by anthropogenic factors, including drinking water treatment, food processing, pharmaceuticals, cosmetics, and consumer products [105,106].

Human exposure to aluminum occurs mainly through food, drinking water, and consumer products. Continuous exposure leads to its accumulation in various tissues, including the bones, liver, kidneys, brain, and mammary glands [107,108]. The level of exposure depends on environmental conditions, dietary habits, and technological processes such as water treatment, which may increase the presence of bioavailable aluminum forms [109].

In the human organism, aluminum has no known essential biological function and has been associated with multiple adverse health effects. Chronic aluminum exposure has been associated with osteomalacia, anemia, and potential neurotoxic effects, including a possible role in neurodegenerative disorders [109,110,111,112,113,114,115,116]. The mechanisms underlying aluminum toxicity remain incompletely understood; however, it has been suggested that aluminum may promote oxidative stress and interfere with cellular processes, particularly in neural tissue [117].

In the maternal organism, aluminum exposure reflects cumulative environmental and dietary intake. Due to its ability to distribute systemically and accumulate in tissues, including mammary glands, aluminum may be transferred into human milk. Although reported concentrations are generally low, this pathway represents a potential source of postnatal exposure for infants [116,117,118].

From the perspective of infant health, exposure to aluminum via breast milk may be relevant due to the immature detoxification and excretory systems in early life. Continuous low-level exposure may potentially contribute to cumulative biological effects, particularly in relation to neurological development. Therefore, environmental and dietary sources of aluminum should be considered as factors influencing maternal body burden and subsequent infant exposure during lactation [116,117].

In summary, although the environmental behavior, toxicokinetics, and biological effects of individual metals differ, several common mechanistic patterns emerge across the analyzed elements. First, environmental contamination of food, water, and air represents the primary upstream determinant of maternal exposure regardless of the specific metal involved. Second, many metals exhibit long-term accumulation within maternal tissues, particularly bone, liver, kidneys, and adipose tissue, creating a persistent internal reservoir that may contribute to exposure even in the absence of ongoing environmental contamination. Third, pregnancy and lactation represent physiologically dynamic periods characterized by tissue mobilization, altered mineral metabolism, and redistribution processes that may facilitate the transfer of toxic elements into human milk.

Importantly, the available evidence suggests that maternal–infant transfer should not be interpreted as a simple linear process, but rather as the result of complex interactions between environmental exposure, maternal physiology, dietary habits, toxicokinetic properties of individual metals, and lactation-related biological changes. Despite differences in chemical speciation and transfer efficiency, metals such as lead, mercury, cadmium, arsenic, chromium, and aluminum collectively illustrate how human milk may function as an interface between environmental contamination and early-life exposure.

Taken together, these findings support the concept that human milk represents not only a nutritional fluid but also a dynamic biomonitoring matrix reflecting the interaction between external environmental conditions and internal maternal body burden. This integrative perspective provides a more comprehensive framework for understanding infant exposure pathways than isolated metal-specific analyses alone.

## 4. Heavy Metals in Human Milk (HM)

Heavy metal concentrations detected in human milk reflect both environmental exposure and maternal body burden. Reported levels vary depending on geographical region, dietary habits, and environmental contamination. Although the reported levels are generally low, they may provide measurable evidence of environmental transfer of toxic elements to the infant during breastfeeding.

Importantly, the occurrence of heavy metals in human milk should not be interpreted as isolated findings related to individual elements alone. Rather, the available evidence indicates that infant exposure results from interconnected environmental, dietary, physiological, and toxicokinetic processes that collectively determine maternal body burden and transfer efficiency during lactation. Consequently, interpretation of heavy metal concentrations requires an integrative perspective that considers both external exposure pathways and internal maternal redistribution mechanisms.

Reported concentrations of heavy metals in human milk should be interpreted with caution due to substantial heterogeneity between studies. To improve comparability and reduce fragmentation of the evidence, key findings from the literature have been additionally synthesized in structured tables summarizing study characteristics, concentration ranges, and geographical variability. Differences in analytical techniques (e.g., ICP-MS vs. AAS), sample preparation, timing of milk collection (colostrum, transitional, mature milk), and the fraction analyzed (whole milk, fat, or aqueous phase) may significantly influence reported values. Therefore, direct comparison between studies is often limited.

### 4.1. Maternal–Infant Exposure to Heavy Metals via Human Milk

#### 4.1.1. Pathways of Heavy Metal Transfer from the Maternal Organism to the Infant—Revised

Infant exposure to heavy metals may begin as early as the fetal stage and continue after birth [119,120,121,122]. During the prenatal period, the primary route of exposure is transplacental transfer, whereas after delivery, breastfeeding becomes an important pathway through which toxic elements may be transmitted [123,124,125]. This suggests that the infant organism may be exposed to heavy metals both during intrauterine development and in the first months of life [124,126,127].

Although the placenta functions as a physiological barrier between the maternal organism and the fetus, numerous chemical substances may still cross it [122,128,129]. Numerous heavy metals can cross the placental barrier via passive or active transport, and their transfer depends, among other factors, on the chemical form of the element, its solubility, and its affinity for transport proteins [127,128]. Lead (Pb) and mercury (Hg), particularly in the form of methylmercury, are considered to cross the placenta relatively efficiently. Methylmercury exhibits high bioavailability and a strong ability to bind to amino acids and plasma proteins [128,130,131,132]. It may be transported across the placenta via amino acid transporters, potentially resulting in fetal blood concentrations comparable to or, in some cases, higher than maternal levels [131,133].

Arsenic (As) is also capable of crossing the placental barrier and may affect fetal development by disrupting metabolic processes, gene expression, and epigenetic mechanisms [131,134]. Prenatal exposure to arsenic has been associated with abnormalities in nervous system development and an increased risk of developmental disorders later in life [123,135,136]. In contrast, cadmium (Cd) appears to be partially restricted by the placenta due to the presence of metallothioneins-metal-binding proteins [123,128,131]. Cadmium may accumulate in placental tissue, thereby limiting its transfer to the fetus; however, under conditions of elevated exposure, a fraction of this element may still enter fetal circulation [119,128,137].

After birth, human milk becomes a major route of postnatal exposure [119,121,122,128,138,139]. Although breastfeeding remains the optimal form of infant nutrition due to its numerous nutritional and immunological benefits, certain toxic substances accumulated in the maternal body may be excreted into milk [119,120,122,128,138,140,141,142]. The transfer of metals into human milk likely involves multiple biological mechanisms. These include passive diffusion across cell membranes, active transport via specialized membrane transporters, and endocytic and exocytic processes enabling the transfer of metal–protein complexes [127,143,144,145]. The relative contribution of these mechanisms varies depending on the physicochemical properties of individual elements. It is also influenced by maternal physiological conditions.

Lead appears to play a particularly important role in this context, as its presence in human milk may reflect not only current environmental exposure but also the mobilization of previously accumulated stores in maternal bone tissue [119,122,128,131,140,146,147,148,149]. During pregnancy and lactation, the demand for calcium increases, which may promote bone resorption [126,127,129,138,149,150,151]. Along with released calcium, previously stored heavy metals, including lead, may enter the bloodstream and potentially contribute to their presence in human milk. As a result, the composition of breast milk may reflect both current and historical exposure to environmental contaminants [119,122,126,129,149,150,152,153].

Importantly, the mechanisms underlying the transfer of heavy metals from the maternal organism to human milk should be interpreted with caution. Although pathways such as bone mobilization, redistribution from soft tissues, and transport through the mammary gland have been proposed, current evidence does not support a uniform or deterministic model applicable to all metals. The relative importance of these pathways likely differs depending on the element, maternal physiological status, and exposure conditions. Therefore, maternal–infant transfer of heavy metals should be considered a dynamic and multifactorial process, characterized by substantial variability and context dependency.

#### 4.1.2. The Role of Human Milk as an Indicator of Environmental Exposure—Subtle Correction

Despite its usefulness in biomonitoring studies, human milk has several important limitations that should be considered when interpreting measured contaminant levels.

Human milk is widely used as a biological matrix in environmental biomonitoring studies [122,126,140,153,154]. Its chemical composition may reflect the level of maternal exposure to various toxic substances, including heavy metals [119,120,122,141,155,156]. The concentration of these elements in milk is influenced by multiple factors. These include maternal diet, lifestyle, place of residence, industrialization level, and occupational exposure [120,142,146,157,158], and their relative contribution may vary across individuals.

For this reason, human milk is often treated as a bioindicator of environmental pollution, as its analysis enables the assessment of population exposure to heavy metals [122,136,152,154,159]. Studies conducted in various regions of the world have demonstrated significant variability in the concentrations of these elements in the milk of lactating women, reflecting differences in environmental contamination, air and water quality, and local dietary habits [120,127,154,160].

The analysis of heavy metal concentrations in human milk may also provide indirect information on both prenatal and postnatal exposure of the infant [120,122,152,161,162]. Particularly valuable are studies on colostrum—the first milk produced in the initial days after childbirth—because its composition is closely linked to maternal metabolism during the final stages of pregnancy [120,122,132,161]. Therefore, the determination of metal content in human milk allows not only the assessment of potential health risks for the infant but also the monitoring of environmental contamination levels in human populations, although interpretation requires consideration of biological and methodological variability [120,122,126,163].

An additional advantage of human milk as a research matrix is its non-invasive collection [129,131,132,136,149]. Unlike blood or tissue samples, milk collection does not require invasive medical procedures for either the mother or the child [129,131,136,149]. Consequently, human milk represents a valuable matrix in environmental epidemiology, enabling simultaneous assessment of maternal body burden of toxic elements and the potential for their transfer to the infant [131,132,153].

However, despite its recognized applicability in biomonitoring studies, human milk should not be interpreted as a direct or stable indicator of environmental exposure. Its composition is highly dynamic and influenced by multiple physiological processes, including the stage of lactation, changes in lipid content, and maternal metabolism. In particular, the concentrations of toxic elements may vary significantly between colostrum, transitional, and mature milk, reflecting not only current exposure but also the mobilization of previously accumulated contaminants from maternal tissues, such as bone and adipose tissue. Furthermore, methodological differences, including sampling protocols, timing of collection, and analytical techniques, may substantially affect reported concentrations and limit comparability between studies. Consequently, human milk should be regarded as a complex, context-dependent biomarker that reflects the interaction between environmental exposure and maternal physiological state, rather than a simple proxy of exposure levels [152,153,154].

#### 4.1.3. Increased Susceptibility of Infants to Toxic Elements

Infants are considered one of the most vulnerable population groups with regard to heavy metal exposure [120,121,128]. This is primarily due to the immaturity of detoxification mechanisms and the intensive development of organs and systems during the first months of life [126,152,163]. Compared with adults, infants have a reduced capacity to metabolize and eliminate toxic substances [131,164].

Hepatic enzyme systems responsible for detoxification, including cytochrome P450 enzymes, do not reach full activity during the first months of life [128,152]. As a result, the biotransformation of many toxic compounds occurs much more slowly than in adults [155]. At the same time, renal excretory function is also immature, limiting the elimination of heavy metals via urine [164,165,166]. Consequently, toxic elements may remain in the infant’s body for longer periods and more readily accumulate in tissues [127,137].

Rapid organ development may further increase infant susceptibility to heavy metals, particularly the central nervous system [119,167,168]. During early life, the brain undergoes intensive processes of growth, neuronal differentiation, and synaptogenesis [119,137]. At this developmental stage, even relatively low exposure levels may interfere with normal brain development [137,138,169].

It is also important to note that the blood–brain barrier in infants is more permeable than in adults [155,164,170]. This facilitates the penetration of metals such as lead and methylmercury into nervous tissue, leading to neurotoxic effects [127,128,131]. Exposure to heavy metals during early childhood may result in cognitive impairments, delayed psychomotor development, and alterations in immune system function [123,127,128,146,169,171].

Infants also exhibit a higher gastrointestinal absorption capacity for heavy metals compared to adults [161,165]. For instance, lead absorption in infants may be several times higher than in adults [121,122,166,172]. At the same time, infants consume significantly greater amounts of food and fluids per unit body weight, resulting in a higher exposure dose to potential contaminants present in the diet [128,152,165,173]. All these factors contribute to the fact that even relatively low concentrations of heavy metals in human milk may pose a significant risk to infant health. However, it should be emphasized that, under typical environmental conditions, exposure levels via human milk are generally low, and breastfeeding remains the recommended and safest feeding practice in the vast majority of cases [138,164,174].

### 4.2. Sources of Heavy Metals in Human Milk

#### 4.2.1. Endogenous Sources

One of the significant sources of heavy metals present in human milk involves processes occurring within the maternal organism, associated with the prior accumulation of these elements in various tissues [121,122,128,140,154,167]. Heavy metals may accumulate in the body over many years as a result of chronic environmental and dietary exposure and may subsequently be mobilized during specific physiological periods such as pregnancy and lactation [119,121,122,128,131,146,162]. During these periods, alterations in mineral metabolism may occur, which can promote the release of previously deposited toxic elements into the bloodstream and potentially contribute to their presence in breast milk [128,149,158].

In addition to bones, heavy metals may also accumulate in soft tissues and adipose tissue [128,136,137,143,155,172,175]. This applies particularly to elements such as cadmium (Cd) and mercury (Hg), especially in the form of methylmercury [136,137,149,155,172]. Cadmium tends to accumulate in parenchymal organs, particularly in the kidneys and liver, where it may persist for extended periods [123,136,155,173]. Mercury, on the other hand, may accumulate in various tissues, including adipose tissue [128,136,137,149]. During lactation, maternal fat reserves are utilized for milk production, which may lead to the mobilization of previously stored compounds [122,144,153,175]. As a result, heavy metals accumulated in tissues may be re-released into the bloodstream and potentially contribute to their presence in human milk [144,153,175].

Taken together, these observations suggest that the concentration of certain metals in breast milk may reflect not only current environmental exposure but also previous long-term exposure of the maternal organism to these elements [125,126,149,153,164,175]. However, the relative contribution of endogenous sources likely varies depending on the specific metal, maternal physiological status, and exposure history, and therefore should be interpreted with caution.

#### 4.2.2. Environmental Sources

An important factor influencing the presence of heavy metals in human milk is the wide range of environmental sources through which these elements enter the maternal organism [120,121,127]. These contaminants may originate from air, soil, water, and food, and their levels often depend on the degree of industrialization of a given region and local environmental conditions [127,140,150,176].

One of the key sources of exposure is air pollution [127,155]. Emissions from road transport, including vehicle exhaust, may introduce significant amounts of heavy metals such as lead and cadmium into the atmosphere [122,127,157,160]. Particularly high concentrations of these elements may occur in regions where leaded gasoline was used in the past [120,122,150,155]. Additional sources include particulate matter generated from tire wear and industrial emissions [127,150]. Metals present in the atmosphere may be inhaled and subsequently enter the bloodstream, increasing the overall body burden in the maternal organism [120,127,137].

Industrial and mining activities are also recognized as major sources of heavy metal contamination [120,127,137]. Women living near mines, smelters, metallurgical plants, or electronic waste incineration facilities are often exposed to higher levels of metals such as lead, mercury, cadmium, arsenic, and nickel [120,122,127,162]. In such regions, metals may enter the environment through industrial emissions, soil contamination, and water pollution, thereby increasing the risk of accumulation in the human body [127,177].

Agriculture also plays a significant role in the dissemination of heavy metals [127]. The use of phosphate fertilizers containing cadmium impurities may lead to the gradual enrichment of soils with this element [127,169,178]. Similarly, certain pesticides may contain arsenic compounds that enter the agricultural environment [127,170,178]. Additionally, irrigation with contaminated water may promote the accumulation of heavy metals in soil and subsequently in crops [127,178]. As a result, these elements may enter the food chain, increasing the risk of human exposure through the consumption of contaminated food [176,177,179].

Dietary intake represents one of the major exposure pathways for heavy metals [120,127,169]. The consumption of fish and seafood is particularly relevant, as these products may contain significant amounts of mercury and arsenic [120,169,170,176]. Heavy metal contamination may also be present in other food products such as cereals (e.g., rice), leafy vegetables, and root crops, including potatoes, especially if they are cultivated on contaminated soils [159,169,179]. Additional sources of exposure may include canned foods, which in some cases may contain trace amounts of metals originating from packaging materials [120,158,169].

Another important source of exposure is drinking water [120,127,157]. Consumption of water from contaminated wells or water sources may result in increased intake of elements such as arsenic and lead [146,157,169]. Of particular concern are old water supply systems with lead pipes, which may release lead into drinking water [122,140,146,150,175]. Long-term consumption of such water promotes the gradual accumulation of this metal in the maternal organism, which may ultimately lead to its presence in human milk [120,122,146].

Collectively, the studies summarized in Table 1 demonstrate that heavy metal occurrence in human milk is shaped by recurring determinants shared across populations, including environmental contamination, dietary exposure, industrialization level, lifestyle-related factors, and maternal physiological status. Despite substantial regional variability, the available evidence consistently indicates that infant exposure through human milk reflects the interaction between external environmental conditions and internal maternal toxicokinetic processes.

### 4.3. Occurrence of Heavy Metals in Human Milk

#### 4.3.1. Most Commonly Analyzed Metals

The occurrence of heavy metals in human milk appears to reflect complex interactions between environmental exposure, maternal body burden, and physiological processes during lactation. However, the detection of these elements does not necessarily imply direct biological effects or health risk for the infant, as their impact depends on multiple factors, including concentration, chemical speciation, bioavailability, and duration of exposure. Interpretation of reported heavy metal concentrations was conducted with consideration of methodological heterogeneity, including differences in study design, analytical techniques, and sample characteristics, which limit direct comparability across studies.

In most reported cases, the concentrations of heavy metals in human milk remain within or close to ranges considered typical for the general population and below levels associated with clear clinical toxicity. Therefore, the presence of these elements should not be interpreted as evidence of immediate health risk, but rather as an indicator of low-level environmental exposure. Importantly, current evidence consistently indicates that the well-established nutritional and immunological benefits of breastfeeding outweigh the potential risks associated with exposure to trace levels of contaminants.

Studies on the chemical composition of human milk place particular emphasis on the presence of heavy metals, which may be of potential concern for infant health [120,123,127,179]. The most frequently analyzed elements include lead (Pb), mercury (Hg), cadmium (Cd), and arsenic (As) [120,121,126,131,180]. These metals are regarded as important environmental contaminants, and their presence in human milk has attracted attention due to the possibility of infant exposure during breastfeeding [120,121,122,131]. From a public health perspective, these elements are commonly monitored because of their toxic potential, ability to accumulate in the body, and possible effects on the developing nervous and immune systems of the child [121,123,127].

In recent years, however, the range of analyzed elements has expanded [120,158]. Increasingly, studies also include metals such as aluminum (Al), chromium (Cr), manganese (Mn), and nickel (Ni) [120,154,158,180]. Interest in these elements is mainly related to their potential relevance for infant development and early-life exposure assessment [123,127,137]. Some of these elements may participate in biological processes; however, their physiological significance in humans remains incompletely understood [126,137,180,181,182,183].

Particular attention has been paid to manganese and chromium, which under physiological conditions participate in numerous metabolic processes and perform specific biological functions in the human body [123,133,137,179]. At the same time, elevated concentrations of these elements may be associated with adverse effects, especially in infants whose nervous system is undergoing intensive development [123,137,181,182]. Excessive exposure to these elements has been linked to disturbances in nervous system function as well as abnormalities in metabolic processes [133,171,181,182].

Other elements, such as aluminum and nickel, are detected in human milk mainly as a result of environmental exposure [133,140]. They may enter the maternal organism through contact with contaminated water, industrial emissions, or the use of certain kitchen utensils made of materials containing these metals [133,140,146,147]. Consequently, these elements may be detected in human milk and are increasingly included in studies assessing the safety of infant feeding [120,154,158,180]. In addition, differences between metals in terms of toxicokinetics and biological behavior further limit the possibility of generalizing occurrence patterns across elements.

#### 4.3.2. Concentration Ranges Reported in Studies

Heavy metal concentrations in human milk demonstrate substantial interindividual and geographical variability. This variability results primarily from differences in environmental pollution levels, maternal diet, lifestyle, and local environmental conditions.

Reported concentration ranges should be interpreted with caution, as they are influenced not only by environmental exposure but also by methodological differences between studies, including the analytical technique used, sample preparation procedures, timing of milk collection, and the milk fraction analyzed. These factors may substantially influence reported concentrations and limit direct comparison across studies [119,120,121,127,132,154,176].

Under conditions considered typical or relatively low-exposure scenarios, reported concentrations of lead in human milk are often in the range of approximately 2–5 µg/L, while mercury levels are typically around 1.4–1.7 µg/L, based on data from population-based studies. In the case of cadmium, concentrations generally remain below 1 µg/L [123,128,132,143,156,167]. These values generally reflect exposure levels reported in populations without pronounced environmental contamination. These reference ranges suggest that heavy metal concentrations in human milk are generally low under typical environmental exposure conditions. Therefore, interpretation of potential health risk should consider toxicological thresholds, exposure duration, and biological susceptibility rather than the mere detection of contaminants alone [128,143,160].

However, in regions with intensive industrial activity or in heavily environmentally contaminated areas, these values may be considerably higher. Cases of extremely high concentrations of heavy metals in human milk have been reported in the scientific literature [123,128,131,153,154,175,176]. For example, the highest recorded lead concentration reached 1515 µg/L in Turkey, whereas a very high arsenic concentration of 149 µg/L was reported in India. Such values likely reflect highly elevated environmental exposure conditions and may indicate increased infant exposure risk in specific high-risk settings. However, these extreme values should be interpreted with caution, as they likely represent outliers associated with highly exposed populations, localized contamination, or potential methodological differences rather than typical concentrations observed in the general population. Therefore, these findings should not be considered representative of background exposure levels but rather as indicators of site-specific high-risk conditions [131,132,152,173,175,177].

The stage of lactation is also an important factor affecting the level of heavy metals in milk [121,161,184,185]. Studies have shown that colostrum, the first milk produced by the maternal organism after childbirth, often contains higher concentrations of certain metals, such as lead, cadmium, aluminum, and arsenic, compared with mature milk. This phenomenon is likely associated with the higher protein content of colostrum, which may bind metals and facilitate their presence in this milk fraction [121,154,158,161,185,186].

Data from studies conducted in Poland indicate that the mean concentrations of heavy metals in the milk of lactating women may reach approximately 6.33 µg/L for lead and 2.11 µg/L for cadmium [131,137,151,179]. These values suggest that in some cases infant exposure may be higher compared with results obtained in certain Scandinavian countries, where environmental contamination levels are generally lower. Overall, the majority of studies indicate relatively low concentrations of heavy metals in human milk in the general population, whereas markedly elevated values are typically associated with specific environmental, occupational, or dietary exposure scenarios, highlighting the importance of contextual interpretation of exposure levels [154,166,179].

#### 4.3.3. Findings from Different Regions of the World

An analysis of studies conducted in different regions of the world indicates that the levels of heavy metals in human milk are strongly dependent on local environmental conditions, industrial structure, and population dietary habits. In many cases, study findings reflect region-specific exposure patterns and environmental conditions [120,121,127,152].

Several studies from Asian regions, including Iran, China, and Pakistan, have reported relatively elevated lead concentrations in human milk. Mean values in certain studies reached approximately 40 µg/L, indicating a considerable environmental burden of this metal [121,131,170,173]. In regions such as Bangladesh and India, contamination with arsenic represents a particularly serious problem, mainly due to the presence of this element in groundwater used for drinking and irrigation. High environmental arsenic levels translate directly into its presence in the bodies of lactating women and in human milk [127,131,134,173].

Studies conducted in several African regions have also demonstrated elevated concentrations of selected heavy metals in human milk [154]. In Nigeria, studies demonstrated very high levels of lead (approximately 38 µg/L) and cadmium (approximately 29 µg/L) [154,155]. In communities inhabiting mining areas in Ghana, particularly high exposure to mercury and arsenic has been observed, which is associated with mining activities and the presence of these elements in the environment [154,167].

In Europe, the situation is more heterogeneous. Nordic countries such as Norway and Sweden belong to the regions with some of the lowest levels of heavy metal contamination in human milk [154,166,184,187]. In studies conducted in these countries, mercury concentrations were approximately 0.2 µg/kg, while cadmium levels were around 0.06 µg/kg [166]. These low values are attributed mainly to strict environmental regulations and high standards of environmental protection [127,175,184]. By contrast, in the Mediterranean region, including countries such as Spain, Italy, and Greece, higher mercury levels have been observed, which are often associated with high fish and seafood consumption, as these foods may accumulate this element [131,152,154].

Studies conducted in South America have shown considerable variation in heavy metal levels depending on the region [131,187]. In Brazil, cases of high mercury concentrations were reported in the Amazon basin region, which is associated with mining activity, especially gold extraction [130,131]. At the same time, in more urbanized areas of the country, lead levels in human milk may be relatively low [143].

In the Middle East, in countries such as Saudi Arabia and Lebanon, levels of toxic metals in human milk are often considered concerning [121,170,173]. Studies conducted in Lebanon demonstrated a strong association between lead concentration in maternal milk and environmental as well as lifestyle-related factors, particularly maternal tobacco smoking and residence near intensively used agricultural areas [170]. These findings highlight the combined influence of environmental and behavioral factors on heavy metal concentrations in human milk. At the same time, regional comparisons should be interpreted cautiously, because differences between studies may reflect not only environmental contamination but also variation in study design, sample size, lactation stage, and analytical methodology. Consequently, repeated patterns observed across multiple populations are more informative than isolated high-value reports [120,127].

### 4.4. Geographical Variability of Heavy Metal Levels

To improve clarity and facilitate comparison, key findings related to geographical variability are summarized in Table 2.

Reported values represent ranges and representative findings from individual studies and should be interpreted in the context of study design, analytical methods, and population characteristics.

As shown in Table 2, the highest concentrations are consistently associated with specific high-exposure contexts rather than representing typical population levels.

Overall, the comparative evidence indicates that geographical variability in heavy metal concentrations is driven not by single isolated factors but by the combined influence of environmental contamination, industrialization, dietary habits, water quality, and maternal physiological characteristics, which together determine infant exposure patterns during lactation.

The structured comparison presented in Table 2 confirms substantial geographical variability in heavy metal concentrations in human milk. Recurring patterns observed across studies indicate that infant exposure is consistently associated with environmental contamination, industrial activity, dietary habits, water quality, and local socioeconomic conditions [119,120,121,127,133,136,137,141,154,160,166,169,170]. Local environmental conditions and dietary habits may substantially influence infant exposure to metals via human milk [120,127,143,152,169,170].

The structured comparison presented in Table 2 confirms that geographical variability in heavy metal concentrations in human milk is substantial; however, it is also strongly influenced by methodological factors, including sample characteristics, analytical techniques, and lactation stage. Therefore, interpretation of regional differences requires consideration of both environmental exposure and study design, and consistent patterns observed across multiple studies are more informative than isolated high-value reports.

Taken together, these findings indicate that geographical variability in environmental contamination translates directly into differences in infant exposure to heavy metals via human milk, with particularly elevated risks observed in regions affected by industrial activity, mining, or contaminated water sources.

However, interpretation of regional differences remains limited by methodological heterogeneity between studies, including differences in sampling protocols, analytical techniques, and analyzed milk fractions.

#### 4.4.1. Differences Between Industrialized and Agricultural Regions

Studies conducted in various regions of the world indicate clear differences in the profiles of heavy metals in human milk depending on the nature of the environment [120,154]. One example is provided by analyses carried out in the Murcia region of Spain, where areas with different degrees of industrialization were compared [120,146]. In industrial and mining areas, the highest concentrations of aluminum, zinc, arsenic, lead, mercury, and nickel were found [120,146,153,154]. In these regions, the mean concentration of lead in human milk was 5.2 µg/L, whereas the zinc level reached the very high value of 1402.6 µg/L [137,146,158]. By contrast, in agricultural areas of the same region, higher concentrations of manganese, chromium, and iron predominated [120,146,153,154]. This phenomenon is mainly associated with the use of mineral fertilizers and plant protection products, which may introduce specific elements into the soil and aquatic environment and subsequently into the food chain [120,127,146,187].

Even more pronounced differences are observed in regions of intensive mining activity. In Ghana, in communities living near gold mines such as Obuasi and Tarkwa, very high levels of metals in human milk were reported. The mean mercury concentration was 7.61 µg/L, corresponding to a value approximately five times higher than the acceptable levels indicated by public health organizations. At the same time, the mean arsenic concentration reached 26.70 µg/L, while the mean lead concentration was 13.83 µg/L. These findings suggest a strong association between mining activity and increased environmental exposure among lactating women [154,167].

Elevated metal levels were also reported in Chile, in the Arica region, where the presence of polymetallic waste disposal sites led to increased concentrations of boron and arsenic in human milk. In this population, the mean boron level was 270 µg/L, whereas in the control group from Santiago it was only 38 µg/L. Simultaneously, the arsenic concentration reached 0.36 µg/L [147,188]. In China, in the Nanjing region, which is characterized by substantial industrial activity, the mean lead level in human milk was 40.6 µg/L [143,170,175]. Other studies showed that women occupationally exposed to lead had a mean colostrum concentration of this element of 52.7 µg/L, whereas in non-exposed women it was only 4.7 µg/L [131]. These findings confirm that the degree of industrialization and the nature of regional economic activity are key determinants of heavy metal levels in human milk [120,127,154,171].

#### 4.4.2. Comparisons Between Urban and Rural Populations

Differences in heavy metal concentrations are also observed between urban and rural populations, although these relationships are not always straightforward [160,176]. In Turkey, in studies conducted in Ankara, the median concentration of lead in human milk was 20.6 µg/L, and in one case an extremely high value of 1515 µg/L was recorded. Importantly, more than 85% of the analyzed samples exceeded the safety threshold of 5 µg/L [125,175]. However, in some regions of the country, the highest concentrations of lead, cadmium, chromium, nickel, and manganese were observed in rural areas [160,189]. This phenomenon has been explained by the use of traditional copper vessels in households and limited control of drinking water quality [157,160].

Similar trends have been observed in Iran, where the mean concentration of lead in human milk samples collected nationwide was 41.9 µg/L, significantly exceeding levels considered safe [148,173]. In large cities such as Kerman and Tehran, these values reached as high as 53.6 µg/L, which was associated with intense road traffic and emissions related to the combustion of leaded gasoline. In contrast, in rural areas of Iran, higher levels of arsenic and mercury were reported compared with urban populations [121,162].

In Greece, the opposite relationship was observed: lead concentrations were higher in women living in urban areas, where they averaged 0.48 µg/L, whereas in rural areas they reached only 0.15 µg/L [136,185]. In Nigeria, significant differences in zinc concentration were demonstrated between different types of environments. In urban areas, the mean level of this element was approximately 350 µg/L, whereas in suburban areas it was around 140 µg/L [154]. At the same time, in the city of Owerri, the mean lead concentration in human milk reached 38 µg/L [155]. These findings indicate that the nature of environmental exposure may differ substantially between regions and that the impact of urbanization on metal levels in human milk depends on local sources of contamination [176].

#### 4.4.3. Influence of Local Environmental Conditions

Specific environmental conditions of a given region, particularly drinking water quality and population dietary habits, also constitute important factors affecting heavy metal concentrations in human milk [120,131,136,152]. One of the best documented examples is arsenic exposure resulting from groundwater contamination [121,131,141,148,157,159]. In Jordan, the mean concentration of arsenic in human milk was 31.7 µg/L, which was directly associated with natural contamination of water used for drinking [180]. A similar phenomenon has been observed in regions of South Asia, especially in Bangladesh and in West Bengal, India, where arsenic concentrations in the milk of lactating women reached median values ranging from 1.8 to 17 µg/L as a result of the use of contaminated tube wells [127,131,167].

Diet is also an important environmental factor, particularly the consumption of fish and seafood, which may constitute the main source of mercury exposure [120,128,131,136,152,154]. In the Amazon region of Brazil, very high concentrations of total mercury in human milk were reported in women consuming large amounts of fish [130,131]. The mean value was 59.41 µg/L, and the maximum reached as high as 104.1 µg/L. By comparison, in regions with lower fish consumption, such as Minas Gerais, mercury levels were several times lower and did not exceed 0.20 µg/L [131]. These data indicate that dietary patterns may substantially modulate the level of infant exposure to heavy metals [120,146,164].

It is also worth noting that in some countries a clear decline in metal levels in human milk has been observed as a result of the implementation of environmental regulations [140,160,189]. In Sweden, following the ban on leaded gasoline, the concentration of lead in human milk decreased from 17 µg/L in 1989 to approximately 1 µg/L in the years 2000–2009 [184,189]. Similar trends were observed in Hungary, where in Budapest the lead level declined by approximately 90%, from 14.9 µg/L in 1991 to 1.74 µg/L in 2017, as a result of the phase-out of leaded gasoline, modernization of water supply infrastructure, and elimination of lead pipes [175].

Taken together, the available evidence indicates that heavy metal concentrations in human milk result from complex interactions between environmental contamination, dietary exposure, maternal physiology, and regional socioeconomic conditions [120,127,154].

### 4.5. Factors Influencing Heavy Metal Concentrations in Human Milk and Their Changes During Lactation

The concentrations of heavy metals in human milk are influenced by a range of environmental, physiological, and behavioral factors related both to the maternal organism and to maternal lifestyle and diet [120,127,190]. In contrast to the general pathways of environmental exposure described earlier, the factors presented in this section refer to potential determinants of metal levels in milk and to changes in their concentrations over the course of lactation [120,140,154]. Available evidence suggests that the elemental composition of human milk reflects a complex interaction between current environmental exposure and long-term accumulation of metals in the maternal body [120,154,161]. However, the relative contribution of individual factors remains difficult to quantify and may vary substantially between individuals and study settings.

In addition, milk composition is not static—it changes dynamically throughout successive stages of lactation, which may also influence the levels of toxic metals and trace elements delivered to the infant [154,157,161,174,190]. These changes should therefore be interpreted in the context of both physiological variability and methodological differences between studies.

#### 4.5.1. Dietary Factors

Maternal diet appears to be one of the key factors influencing heavy metal concentrations in human milk, since many elements may enter the body through food and drinking water [120,140,164,169,191]. Of particular importance is the consumption of fish and seafood, which constitute the main source of exposure to mercury and arsenic [120,147,154,169,176]. These metals undergo biomagnification in the food chain, meaning that their concentration increases with the trophic level of aquatic organisms [120,136,142,169,173]. Studies conducted in Norway demonstrated that high seafood consumption leads to a significant increase in mercury levels in human milk, with the mean concentration of this element being approximately 42% higher in women who regularly consumed marine products [166]. Similar associations were observed for other categories of fish. Consumption of lean fish was associated with an increase in mercury concentration of approximately 32%, and elevated levels of this metal were also observed in women consuming halibut and shellfish [120,121,152,158,166].

The consumption of plant products and cereals is also of considerable importance [150,158,162,169,170]. In many regions of the world, rice and cereal products constitute the main source of arsenic in the human diet [136,141,147,159,170]. Accordingly, frequent consumption of these products correlates with higher arsenic levels in human milk [158,164,169,173]. Similarly, cadmium shows a clear association with plant-based diets, particularly with the consumption of leafy vegetables, legumes, and potatoes [150,158,168,169]. Some studies have also demonstrated a strong correlation between cadmium levels and the consumption of processed potato-based products such as chips, with a correlation coefficient of r = 0.502 [158].

The consumption of animal-derived products may also affect metal levels in human milk [154,158,164,169]. It has been shown that the consumption of certain cheeses, for example feta cheese, may be associated with higher lead concentrations. At the same time, there are data indicating an inverse relationship between red meat consumption and lead levels in milk [158,169]. This mechanism most likely results from the presence of iron in meat, which competes with lead for the same membrane transporters in the intestine, particularly the DMT1 transporter. High iron intake may therefore reduce lead absorption into the maternal organism [127,143,190].

An important dietary component is also the source of drinking water [120,154,159,169,188]. Consumption of water obtained from wells or private water sources may significantly increase the risk of lead and arsenic presence in human milk [140,146,154,158,159]. In many regions of the world, groundwater naturally contains elevated concentrations of these elements or becomes contaminated as a result of industrial and agricultural activities [127,134,138,141,170].

#### 4.5.2. Lifestyle-Related Factors

Maternal lifestyle factors may also contribute substantially to heavy metal levels in human milk. One of the most consistently reported factors is tobacco smoking [120,128,131,158,179]. Tobacco contains considerable amounts of cadmium, with the concentration in a single cigarette being approximately 0.1–0.2 µg [120,146]. Cadmium is readily absorbed through the respiratory tract and subsequently accumulates in the body [120,137,138,179]. As a result, women who smoke exhibit clearly higher concentrations of this metal in milk [128,170,179]. Studies indicate that the cadmium level in the milk of smoking mothers may be 37% to as much as 300% higher compared with non-smoking women [161,175]. Importantly, passive smoking may also increase exposure to metals [120,150]. It has been shown that staying in a smoke-filled environment for more than 15 min per day may lead to increased concentrations of aluminum and cadmium in human milk [192].

Occupational exposure is also an important risk factor [120,128,193]. Women working in the metallurgical or chemical industries or in agriculture, where pesticides are used, may be exposed to elevated levels of heavy metals [121,127,193]. Occupational exposure to metals leads to their accumulation in the body, which may subsequently result in higher concentrations in milk during lactation [121,127,128].

Residential environments may also influence exposure patterns. Women living near roads with heavy traffic are more exposed to lead. The sources of this metal include primarily vehicle emissions as well as dust containing metal particles originating from tire wear and brake system components [120,125,137,157,158,160,175,177].

#### 4.5.3. Biological Factors

Heavy metal levels in human milk are also influenced by biological factors related to the maternal organism [127,153,158,159,175]. One of these is maternal age. A positive correlation between maternal age and lead concentration in milk has been demonstrated [120,136,146,158,161,162,175,179,193]. This results from the fact that lead accumulates in bone tissue throughout life. During lactation, bone metabolism increases and stored elements are mobilized, which may lead to their release into the bloodstream and subsequently into milk [121,122,129,137,140,148,149,151,158,159,161,162,174,175,190,191,194]. In studies conducted in Poland, the mean lead concentration increased from 4.76 µg/L in women aged 20–25 years to 7.41 µg/L in women aged 36–40 years [175,179].

Another important factor is the number of previous births, referred to as parity. Studies indicate that primiparous women often have higher concentrations of heavy metals in milk, including lead, cadmium, and mercury, compared with multiparous women [120,121,128,153,154]. This suggests that successive lactation periods may contribute to a gradual reduction in maternal stores of accumulated metals [121,144,146,154].

The general health status of the woman is also important [120,151,155,158,159,177]. In particular, iron-deficiency anemia is of major relevance. In such cases, the body increases the absorption of metals from the gastrointestinal tract through transporters responsible for iron transport [125,132,151,155,158,169]. Since lead uses the same transport mechanisms, iron deficiency may lead to increased absorption of this metal and, consequently, to higher lead concentrations in human milk [125,128,131,132,169].

#### 4.5.4. Other Sources of Exposure

In addition to diet, lifestyle, and biological factors, there are also other sources of exposure to heavy metals that may affect their levels in human milk. One of these is dental amalgam fillings used in dentistry. So-called “silver fillings” contain mercury, which may be gradually released into the body [121]. Studies have shown that the number of such fillings is a significant predictor of mercury concentration in milk [149,166]. In women with at least one amalgam filling, mercury levels in milk were approximately three times higher than in women without such fillings [121].

Certain cosmetic products may also be relevant. It has been shown that the use of lipsticks and color cosmetics may be associated with increased lead levels in human milk [120,141,148]. In one study, a positive correlation was demonstrated between the use of these products and lead concentration in milk, with a regression coefficient of β = 0.32 [141,148].

By contrast, the use of dietary supplements containing vitamins and minerals may have a protective effect [126]. Supplementation with calcium and iron may reduce the absorption of cadmium and lead in the gastrointestinal tract and may also decrease the mobilization of these elements from bone into the bloodstream [127,174].

#### 4.5.5. Changes in Element Concentrations During Lactation

The concentrations of metals and trace elements in human milk undergo significant changes during successive stages of lactation. The highest levels of many toxic metals are observed in colostrum, that is, in the first phase of lactation immediately after childbirth [161,185]. This applies primarily to lead, arsenic, aluminum, and mercury. During the following weeks and months of lactation, their concentrations usually decline markedly [121,161]. Studies conducted in Taiwan showed that the levels of lead, aluminum, and arsenic may decrease by as much as 75–90% within the first two months of breastfeeding [161,175]. This decline is mainly associated with changes in milk composition, especially with the reduction in protein content, as proteins are the main ligands binding metals [121,161].

Cadmium constitutes an exception, as its pattern of change during lactation differs from that of other metals. Many studies have shown that its concentration remains relatively stable or may even increase in mature milk, reaching the highest values between the fourth and sixth months of lactation [179]. The mechanism of this phenomenon may be related to active cadmium transport in mammary gland cells and to its competition with zinc and calcium for common membrane transporters [142,144].

At the same time, lactation is associated with a marked decline in the concentrations of many biologically important trace elements, such as zinc, copper, selenium, and iron [184,195]. Among these, zinc shows the most rapid decrease [195]. Studies have demonstrated that its concentration may decline from approximately 4.9 mg/L in colostrum to about 2.9 mg/L in transitional milk [185]. This decrease is regarded as a physiologically programmed process and is largely independent of the mother’s current mineral status [157]. These changes reflect the adaptation of milk composition to the changing needs of the developing infant [195].

### 4.6. Analytical Methods Used to Determine Heavy Metals in Human Milk

The analysis of heavy metals in human milk represents one of the more demanding analytical challenges in biomonitoring studies [131,153,165]. This is primarily related to the complex nature of the biological matrix, which is characterized by a high content of fats, proteins, and other organic compounds that may interfere with analytical determination and affect recovery if sample preparation is not properly standardized [195,196,197]. An additional limitation arises from the fact that most heavy metals occur in human milk at very low concentrations, often at trace levels close to the detection limits of commonly applied techniques [126,175,184].

However, a key issue in this context is not only analytical sensitivity, but also the lack of harmonized sample preparation and analytical protocols, including differences in lipid removal, digestion procedures, and the fraction of milk analyzed (e.g., whole milk vs. aqueous phase) [131,153,165,195,196,197]. These methodological inconsistencies may significantly influence reported concentrations and contribute to variability between studies [126,175,184]. Moreover, the composition of human milk changes dynamically across lactation stages, which may alter metal-binding properties and further affect analytical outcomes [131,153,165].

Another important challenge is the limited availability of certified reference materials specific to human milk, which restricts method validation and reduces comparability of results across laboratories. Consequently, interlaboratory variability remains substantial, even when similar analytical approaches are used [131,170,198]. Although highly sensitive techniques enable the determination of elements at very low concentrations (ppb or ppt levels), the main limitation in current research lies not in detection capability itself, but in the lack of methodological standardization and matrix-specific validation, which hampers the interpretation and comparison of biomonitoring data [172,198,199].

Therefore, caution should be exercised when comparing reported concentrations of heavy metals in human milk across studies, as methodological differences may substantially affect the observed values.

#### 4.6.1. Methods Used in Laboratory Analyses (ICP-MS, AAS, GFAAS, CV-AAS)

One of the most frequently used methods in the analysis of heavy metals in human milk is inductively coupled plasma mass spectrometry (ICP-MS) [121,140,169,170,196,198]. This technique is currently regarded as the “gold standard” in trace element biomonitoring [121,136,170,196]. Its greatest advantage is the ability to determine a very large number of elements simultaneously—often more than fifty—in a single measurement cycle lasting only a few minutes [167,198]. ICP-MS is characterized by a very wide dynamic range and exceptionally low detection limits, which makes it possible to analyze elements present in trace amounts [121,164,175,184,198]. This method enables the detection of metals in the picogram range or at parts-per-billion and parts-per-trillion levels, which is particularly important in the analysis of human milk, where the levels of many toxic elements are very low [172,175,198,199].

Another widely used technique is atomic absorption spectrometry (AAS). This is a classical analytical method that for many years served as a fundamental tool for the determination of metals in biological and environmental samples [131,169,170,198,199]. In this technique, the measurement is based on determining the amount of light absorbed by free atoms of an element in the gaseous state [198,199]. Depending on the method of sample atomization, several variants of this method can be distinguished [131,198]. One of them is flame atomic absorption spectrometry (FAAS) [121,131,154,161,198]. This method is relatively simple, rapid, and cost-effective; however, its sensitivity is limited, which means that in many cases it does not allow the determination of very low metal concentrations in milk without prior sample preconcentration [121,175,198].

Much greater sensitivity is offered by graphite furnace atomic absorption spectrometry (GFAAS), also referred to as electrothermal atomic absorption spectrometry (ET-AAS) [131,161,169,198]. In this method, the sample is heated in a graphite furnace to very high temperatures, allowing efficient atomization of the analyzed elements [198,199]. As a result, detection limits are 20- to 200-fold lower than in flame atomization [121]. In practice, this method is particularly useful for the determination of metals such as cadmium and lead, which often occur in human milk in very small amounts [121,161,198,199,200]. An additional advantage of this technique is the possibility of analyzing very small sample volumes, which is important in biological studies [198,199,200].

A specialized variant of atomic absorption spectrometry is cold vapor atomic absorption spectrometry (CV-AAS), a method intended exclusively for the determination of mercury [119,121,131,169,198]. This technique makes use of the unique property of mercury to readily pass into the gaseous phase at room temperature [167,198]. During analysis, mercury is reduced to its elemental form and then transported as vapor into the measurement chamber [119,164,167,198]. This enables highly selective and sensitive determination of this element even at very low concentrations [198].

#### 4.6.2. Importance of Methods for the Determination of Trace Elements at Low Concentrations

In the analysis of heavy metals in human milk, the ability of analytical methods to determine elements at very low concentrations is of crucial importance. In many cases, the levels of toxic metals in this biological matrix are close to the detection limits of many classical analytical techniques [131,196,198]. Therefore, the use of methods characterized by high sensitivity, low detection limits, and good repeatability is particularly important [131,198].

The best analytical performance is achieved by ICP-MS, which enables detection of elements at extremely low concentrations [140,172,198,199]. For example, the detection limit for lead in this method may be approximately 0.0051 µg/L, for cadmium about 0.0100 µg/L, and for mercury about 0.0060 µg/L [182]. Such high sensitivity makes this method particularly useful in biomonitoring studies and in the analysis of biological samples, where even small differences in concentration may be of considerable toxicological relevance [153,172,196].

In GFAAS, detection limits are higher than in ICP-MS, but they are still sufficiently low to allow the determination of trace metals in human milk [121,131,198]. They usually range from approximately 0.01 to 1 µg/L, which enables reliable analysis of elements such as lead and cadmium. The high sensitivity of this method makes it frequently used in studies focused on individual metals [198,199].

An important quality parameter of analytical methods is analyte recovery, that is, the extent to which the applied procedure allows the recovery of the actual amount of an element from the sample [164,165,195]. In properly validated methods, recovery should generally fall within the range of approximately 90–110% [139,164,165]. This means that the method is capable of reproducing the true concentration of the element with minimal analytical error [164,165]. Another important indicator is measurement precision, often expressed as the coefficient of variation (CV) [148,165,195]. In high-quality analyses, this value should generally not exceed 5–10%, indicating good repeatability of results [148,164,165,180,195,196].

The modern approach to the analysis of metals in biological samples also includes the possibility of determining the chemical forms of elements, that is, speciation [165,198]. For this purpose, hyphenated techniques such as HPLC-ICP-MS are used, enabling separation of different chemical forms of a metal prior to its determination [198]. This is particularly important in the case of elements such as arsenic and mercury, because their toxicity depends largely on their chemical form [123,170,198]. For example, inorganic arsenic is considerably more toxic than its organic forms, whereas methylmercury exhibits much higher bioavailability and a greater tendency to accumulate in the body than inorganic mercury [170,198].

As a result, the use of appropriately sensitive and selective analytical methods is a key element in the reliable assessment of infant exposure to heavy metals through breast milk [127,198]. Owing to modern spectrometric techniques, it is possible not only to determine very low concentrations of these elements but also to accurately assess their chemical forms and potential toxicological risk. This methodological variability is one of the main reasons why reported concentrations of heavy metals in human milk differ considerably across studies. For this reason, interpretation of the available evidence should consider not only the reported values themselves, but also the analytical approach, sample handling, and timing of collection [198].

## 5. Public Health Implications

From a public health perspective, the available evidence allows for several practical recommendations aimed at reducing infant exposure to heavy metals while maintaining the well-established benefits of breastfeeding.

Pregnant and lactating women should limit consumption of high-risk foods, particularly large predatory fish, which constitute a major source of mercury exposure, and ensure a balanced diet rich in essential nutrients such as iron and calcium, which may reduce the absorption of toxic metals such as lead [120,127,143,169,190]. Attention should also be given to drinking water quality, especially in regions where contamination with arsenic or lead is known to occur [146,157,169]. Smoking cessation is strongly recommended, as tobacco represents a significant source of cadmium exposure and may substantially increase its concentration in human milk [120,146,179].

From a regulatory perspective, it should be emphasized that no universally established safety thresholds exist for heavy metals specifically in human milk, and risk assessment is generally based on tolerable intake levels defined for infants by organizations such as the World Health Organization and the European Food Safety Authority [127,169].

Although human milk is widely used as a biomonitoring matrix in population studies, its application at the individual level remains limited and rarely translates into direct clinical recommendations [122,126,153].

Importantly, despite the presence of contaminants, breastfeeding remains the preferred method of infant feeding, as infant formulas may also contain measurable levels of heavy metals originating from raw materials and processing. Therefore, efforts should focus primarily on reducing environmental and dietary exposure rather than discouraging breastfeeding [120,154,158].

## 6. Conclusions

The available evidence consistently indicates that human milk remains the gold standard for infant nutrition, providing not only essential nutrients but also a wide range of bioactive compounds necessary for proper child development. At the same time, as a biological fluid reflecting maternal environmental exposure, it may represent a potential route of infant exposure to heavy metals and other xenobiotics. This duality—high biological value combined with the possibility of contaminant transfer—constitutes an important area of discussion and requires a balanced and critical interpretation.

The presence of heavy metals in human milk appears to be associated with the cumulative nature of environmental exposure and the mobilization of maternal body reserves, particularly during lactation. Processes such as bone resorption and adipose tissue utilization may contribute to the release of previously stored toxicants, suggesting that milk composition may reflect both current and historical exposure of the maternal organism. This observation highlights the importance of considering long-term exposure patterns when designing preventive strategies.

A major strength of the available studies lies in their global scope and the ability to identify geographical variability. Available evidence suggests that heavy metal concentrations in human milk differ across regions and are influenced by factors such as the level of industrialization, environmental quality, diet, and lifestyle. These findings are relevant from a public health perspective, as they may help identify populations potentially at higher risk of exposure. Moreover, human milk can serve as a valuable, non-invasive biomonitoring matrix for assessing environmental exposure in both mothers and infants.

However, despite the growing number of studies, several important methodological limitations remain. Many analyses are based on cross-sectional designs with relatively small sample sizes, which limits the generalizability of the findings. In addition, the lack of standardization in analytical methods, as well as in sampling and sample preparation protocols, complicates direct comparison between studies. Furthermore, potential confounding factors, such as diet, socioeconomic status, and co-existing environmental exposures, are not always adequately controlled, which may affect the interpretation of results. Another limitation is the relatively small number of longitudinal studies that would allow for the assessment of temporal changes in metal concentrations and their long-term effects on child health. Although the toxicity of metals such as lead and methylmercury is well documented, there is still limited evidence defining safe exposure thresholds for breastfed infants, particularly in the context of chronic low-dose exposure, which introduces uncertainty in risk assessment.

It should also be emphasized that many studies focus primarily on selected metals (Pb, Cd, Hg, As), while other potentially relevant elements and interactions between them are less frequently considered. In reality, exposure is multi-component in nature, and potential effects may result from combined or synergistic interactions between different compounds.

Future research should aim to address these limitations by standardizing analytical approaches, improving exposure assessment, and providing more longitudinal data on infant health outcomes. The development of highly sensitive analytical techniques (e.g., ICP-MS), including the assessment of chemical speciation, is of particular importance. Additionally, well-designed cohort studies are needed to link exposure levels with specific health outcomes in children.

From a public health perspective, it is important to emphasize that, despite the presence of contaminants, the benefits of breastfeeding are widely recognized to outweigh the potential risks in the general population. Therefore, efforts may be directed toward reducing environmental and dietary sources of contamination; however, the effectiveness of such strategies in lowering infant exposure via human milk requires further investigation. Importantly, the current evidence is based primarily on observational and biomonitoring studies, while data from controlled intervention studies remain limited.

The available evidence indicates that environmental and dietary exposure pathways are reflected in measurable concentrations of heavy metals in human milk; however, the relationship between exposure, transfer, and potential health effects remains complex and context-dependent.

Overall, human milk should be considered not only as the nutritional gold standard for infants, but also as a sensitive indicator of maternal exposure and a biologically relevant pathway of early-life contact with environmental contaminants.

## Figures and Tables

**Table 1:** Summary of selected studies on heavy metals in human milk, including study characteristics, analytical approaches, and reported findings.

Region/Country	Metal(s) Analyzed	Key Findings and Exposure Implications	Study Context	Analytical Method	Reference
Nigeria	Pb, Cd, Zn, Fe, As, Hg	Elevated levels of toxic and trace metals indicating increased environmental exposure	Local population study	AAS/ICP-based methods	Olujimi et al., 2023 [142]
Nigeria	Pb, Cd, Cr, Cu, Zn, Fe, As, Hg	Distinct exposure profiles associated with industrial and agricultural environments	Postpartum mothers	ICP-MS	Ekeanyanwu et al., 2020 [155]
Iran	Multiple metals	Infant exposure influenced by combined environmental and physiological factors	Exposure modeling study	Modeling approach	Samiee et al., 2019 [148]
Spain (Murcia)	Pb, As, Hg, Ni, Zn	Human milk reflects maternal and infant exposure burden	Environmental exposure	ICP-MS	Motas et al., 2021 [146]
Brazil	Multiple metals	Correlation between milk, soil, and water contamination	Environmental study	AAS/ICP	Cardoso et al., 2014 [143]
Croatia	Pb, Cd, Hg, others	Human milk serves as a biomonitoring matrix of maternal and infant exposure	Population-based study	ICP-MS	Grzunov Letinić et al., 2016 [149]
Turkey	Multiple metals	Regional biomonitoring confirms the presence of multiple exposure pathways	Local population study	ICP-MS	Kılıç Altun et al., 2018 [157]
Cyprus	Pb, Cd, others	Environmental contamination contributes to detectable metal burden in human milk	Regional study	AAS	Kunter et al., 2016 [156]
Poland	Essential and toxic elements	Human milk as an indicator of infant exposure burden	Biomonitoring study	ICP-MS	Bzikowska-Jura et al., 2024 [158]
Global	Multiple metals	Integrated evidence of multifactorial infant exposure pathways	Review study	-	Onyena et al., 2024 [154]
Global	Multiple contaminants	Recent evidence highlighting widespread environmental contamination patterns	Review study	-	Serreau et al., 2024 [153]
Global	Multiple contaminants	Broad evidence supporting the biomonitoring role of human milk	Review study	-	Bernasconi et al., 2022 [152]
Nigeria	Multiple metals	Maternal health status may influence contaminant burden in human milk	Clinical study	ICP-MS/AAS	Philip-Slaboh et al., 2023 [151]
Iran	Trace elements	Lifestyle-related factors contribute to variability in metal concentrations	Lifestyle-related study	ICP-MS	Mansouri et al., 2023 [150]
Brazil	Essential and toxic elements	Processing and sample characteristics may influence contaminant assessment	Preclinical study	ICP-MS	Oliveira et al., 2020 [147]

**Table 2:** Geographical variability of heavy metal concentrations in human milk across different regions and exposure contexts.

Region/Country	Exposure Context	Metal(s) Analyzed	Reported Concentration Range (µg/L)	Key Observation	References
Global (reported background levels)	General population	Pb, Hg, Cd	Pb: 2–5; Hg: 1.4–1.7; Cd: <1	Typical background exposure levels in non-contaminated populations	[123,128,132,143,156,167]
Turkey (Ankara)	Urban/mixed exposure	Pb	Median: 20.6; max: 1515	Extremely high outlier values; majority of samples exceeded safety threshold	[125,175]
India	Environmental contamination	As	up to 149	Very high arsenic levels indicating severe environmental exposure	[131,132,152,173,175,177]
Iran (national/urban)	Urban/traffic-related	Pb	Mean: 41.9; up to 53.6	Elevated Pb linked to urban pollution and traffic emissions	[148,173]
China (Nanjing)	Industrial	Pb	Mean: 40.6	High levels associated with industrial activity	[143,170,175]
Ghana (mining areas)	Mining	Hg, As, Pb	Hg: 7.61; As: 26.7; Pb: 13.83	Strong influence of mining activity on exposure levels	[154,167]
Nigeria	Urban/environmental	Pb, Cd	Pb: ~38; Cd: ~29	High levels in urban populations	[154,155]
Spain (Murcia—industrial)	Industrial/mining	Pb, Zn, As, Hg, Ni	Pb: 5.2; Zn: 1402.6	Elevated multi-metal exposure in industrial regions	[120,146,153,154]
Spain (Murcia—agricultural)	Agricultural	Mn, Cr, Fe	Not numerically specified	Dominance of agriculturally derived elements	[120,146,153,154]
Brazil (Amazon)	High fish consumption/mining	Hg	Mean: 59.41; max: 104.1	Very high Hg due to dietary intake (fish)	[130,131]
Brazil (urban areas)	Urban	Pb	Low (not specified)	Lower Pb levels in less contaminated areas	[143]
Scandinavia (Norway, Sweden)	Low pollution	Hg, Cd	Hg: ~0.2 µg/kg; Cd: ~0.06 µg/kg	Lowest reported levels due to strict environmental regulations	[166,184,187]
Jordan	Drinking water contamination	As	Mean: 31.7	Direct link with contaminated groundwater	[180]
Bangladesh/India (rural)	Groundwater exposure	As	Median: 1.8–17	Chronic exposure via contaminated water	[127,131,167]

## Data Availability

No new data were created or analyzed in this study.
